# Supplementary material for: Abnormal Calcium Handling in Atrial Fibrillation Is Linked to Changes in Cyclic AMP Dependent Signaling
Source: Cells. 2021 Nov 5;10(11):3042. doi: 10.3390/cells10113042 (PMC8616167; doi:10.3390/cells10113042)
Supplement: Supplementary file 1 [file cells-10-03042-s001.zip › cells-1366341-supplementary.pdf]

Supplementary figures

Abnormal calcium handling in atrial fibrillation is linked to changes in cyclic AMP dependent signaling

Franziska Reinhardt [1,2 †], Kira Beneke [2,3 †], Nefeli Grammatica Pavlidou[2,3], Lenard Conradi[1], Hermann Reichenspurner[1,3], Leif Hove-Madsen[4] and Cristina E. Molina [2,3*]

[1] Department of Cardiovascular Surgery, University Heart & Vascular Center Hamburg UKE, Germany
[2] German Center for Cardiovascular Research (DZHK), partner site Hamburg/Kiel/Lübeck, Hamburg, Germany
[3] Institute of Experimental Cardiovascular Research, University Medical Center Hamburg-Eppendorf (UKE), Germany
[4] Biomedical Research Institute Barcelona, IIBB-CSIC and IIB Sant Pau, Hospital de la Santa Creu i Sant Pau, Barcelona, Spain
* Correspondence: c.molina@uke.de; Tel.: 49-40741057095
† These authors contributed equally to this work

Correspondence:
Cristina E. Molina
Zentrum für Experimentelle Medizin
Institut für Experimentelle Herz-Kreislaufforschung
Universitätsklinikum Hamburg-Eppendorf
Martinistrasse 52, W23
20246 Hamburg
Germany
Tel: +49 (0)40 7410-57095
Fax: +49 (0)40 7410-40180
Email: c.molina@uke.de

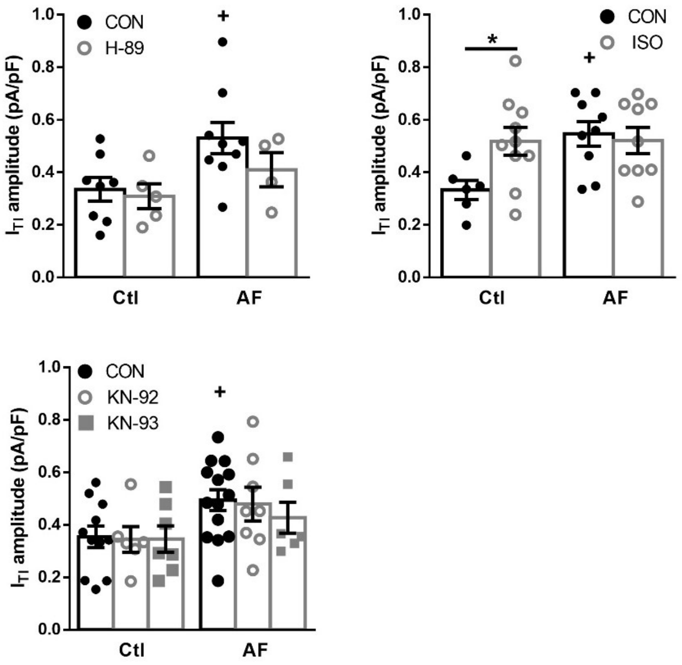

Supplementary Figure S1. Effects of H-89, isoproterenol (ISO) and Ca^{2+}/Calmodulin-dependent protein kinase II (CaMKII) inhibition with KN-92 and KN-93 on I_{TI} amplitude. Average effects of protein kinase A (PKA) inhibition with H-89 (top left panel), PKA stimulation with ISO (top right panel), and CaMKII inhibition (bottom panel) with KN-92 (the inactive CaMKII inhibitor) and KN-93 in sinus rhythm (Ctl) and atrial fibrillation (AF) patients. Significant differences between treatments are indicated with * and between groups with +.

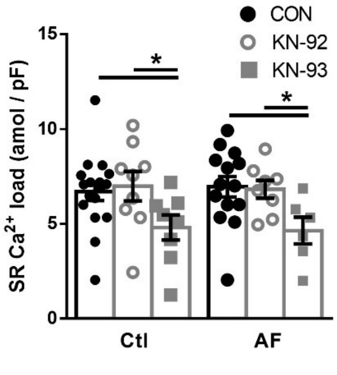

Supplementary Figure S2. Effects of Ca^{2+}/Calmodulin-dependent protein kinase II (CaMKII) inhibition on sarcoplasmic reticulum (SR) Ca^{2+} load. Average effect of CaMKII inhibition with KN-92 (the inactive CaMKII inhibitor) and KN-93 on SR Ca^{2+} load in sinus rhythm (Ctl) and atrial fibrillation (AF). Significant differences between treatments are indicated with *.
